# Application of Zebrafish Models in Inflammatory Bowel Disease

**DOI:** 10.3389/fimmu.2017.00501

**Published:** 2017-05-03

**Authors:** Li Hanyang, Liu Xuanzhe, Chen Xuyang, Qiu Yujia, Fu Jiarong, Shen Jun, Ran Zhihua

**Affiliations:** ^1^Division of Gastroenterology and Hepatology, Key Laboratory of Gastroenterology and Hepatology, Ministry of Health, Inflammatory Bowel Disease Research Center, Shanghai, China; ^2^Renji Hospital, School of Medicine, Shanghai Jiao Tong University, Shanghai, China; ^3^Shanghai Institute of Digestive Disease, Shanghai, China

**Keywords:** zebrafish, inflammatory bowel disease, murine model, pathogenesis, drug screening

## Abstract

Inflammatory bowel disease (IBD) is a chronic, recurrent, and remitting inflammatory disease with unclear etiology. As a clinically frequent disease, it can affect individuals throughout their lives, with multiple complications. Unfortunately, traditional murine models are not efficient for the further study of IBD. Thus, effective and convenient animal models are needed. Zebrafish have been used as model organisms to investigate IBD because of their suggested highly genetic similarity to humans and their superiority as laboratory models. The zebrafish model has been used to study the composition of intestinal microbiota, novel genes, and therapeutic approaches. The pathogenesis of IBD is still unclear and many risk factors remain unidentified. In this review, we compare traditional murine models and zebrafish models in terms of advantages, pathogenesis, and drug discovery screening for IBD. We also review the progress and deficiencies of the zebrafish model for scientific applications.

## Introduction

Inflammatory bowel disease (IBD) is a general description of gastrointestinal tract diseases that show a chronic or recurring immune response and inflammatory symptoms. The two most common types of IBD are ulcerative colitis (UC) and Crohn’s disease (CD). In CD, the inflammation affects the whole digestive tract, whereas in UC, only the colon is involved. Both these diseases are characterized by an abnormal response by the immune system, compromising diarrhea or rectal urgency, bleeding, abdominal pain, constipation, and loss of appetite (1). In addition, IBD can cause a number of non-digestive tract complications. Parenteral complications severely interfere with patients’ quality of life. As a far-ranging disease, IBD can occur at any age and affects both sexes. About 600,000 new cases of IBD are diagnosed in America every year (2). However, the pathogenesis and etiology of IBD remain incompletely understood. Controversy concerning the etiology of IBD exists because of its multifactorial pathogenesis. It is believed that susceptible genes and environmental factors are responsible for the pathogenesis of IBD (2). Both of these factors lead to an abnormal chronic or recurring immune response, resulting in tissue injury with ulceration and bowel inflammation (2). Animal models can help investigators to identify disease mechanisms and progression, and to develop drugs and targeted therapy (3). So far, the most well-established model in IBD is the murine model (3). As a valuable animal model, it has contributed a lot to the understanding of IBD, in terms of the aspects including gut microbiota, immunoreaction, infection, and inheritance. However, as IBD studies have progressed, limitations of the traditional murine model have emerged. For example, the pathological changes in the murine model cannot be determined by continuous observation. The traditional murine model breeds a generation in about 2 months (4). To some extent, this is too slow for active IBD research (5). For genetic studies, the genetic manipulation of the murine models is difficult. As the drug screening research continues, the traditional murine model has become less useful in terms of modeling speed and modeling quantity (6). These defects of the murine model have prompted investigators to find better models to study the pathogenesis and therapeutic approaches for IBD.

## Construction of Zebrafish Models

**Figure 1:**
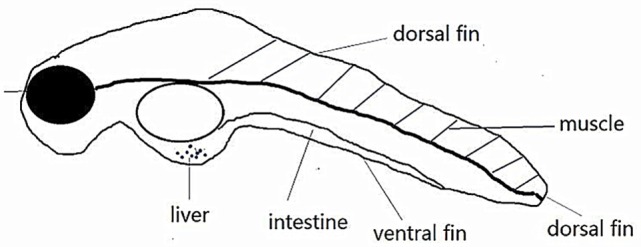
**Schematic diagram of zebrafish internal organs**.

Zebrafish (*Danio rerio*) larvae have emerged as a useful tool to study IBD and gastrointestinal diseases (7). Compared with humans, the zebrafish has a highly similar gastrointestinal system, with a liver, gall bladder, pancreas, and intestinal tract with comparable absorptive and secretory functions (Figure 1) (8, 9).

In addition, a genome alignment showed that zebrafish orthologs exist for over 70% of human genes (10). This high similarity, and a clearer understanding of the critical genomic features, could promote the identification of susceptibility genes of IBD (11). For example, *NOD1* and *NOD2* encode two types of nucleotide-binding oligomerization domain-containing proteins, both of which recognize bacterial molecules and stimulate an immune reaction (11). Thus, zebrafish have the potential to act as a model in studies of the genetic and environmental aspects of IBD pathogenesis.

Zebrafish have already attracted the attention of researchers because of their susceptibility to mutagenesis and the availability of transgenic technology (12, 13). Commonly, the strains used in zebrafish models for IBD studies have included three major types: wild-type strains (WT strains), mutant strains, and transgenic strains. The most common type for scientific study is the AB WT strain, which is usually exposed to a sequence of colitogenic agents, containing live bacteria, bacterial products, and chemicals (12). Notably, *n*-ethyl-*n*-nitrosourea (ENU), an advanced genetic mutagenesis agent, can generate zebrafish mutants and has been used to develop spontaneous intestinal neoplasia models (13). These heterozygous mutations were confirmed to have a relationship with truncated forms of adenomatous polyposis coli (APC). The accumulated effects included an increase of nuclear β-protein and excessive expression of downstream genes such as c-MYC and AXIN2 (13). When the carcinogen 7,12-dimethylbenzanthracene was used in the zebrafish model, the frequency of gastrointestinal disease development increased, especially those involving the hepatic system, bile duct, and intestinal tract (13). APC mutations cause the spontaneous development of intestinal polyps (14–16). Therefore, the APC zebrafish model could be applied to genetic, drug screening, and toxicology studies for other intestinal diseases, including IBD.

In addition to ENU, intestinal damage models are often induced by dextran sodium sulfate (DSS) or trinitrobenzene sulfonic acid (TNBS) (17). Chemical models of enterocolitis are induced conveniently by oxazolone, lipopolysaccharide (LPS), and glafenine. Agents and chemical models cause non-specific intestinal defects. Chemical genetic methods can also manipulate the host response to injury, which is convenient for use in IBD models (17). The effect of certain chemicals can be analyzed in a specific biological system, and small molecules are then used to disturb the biological processes directly. Compared with the pure chemical model, chemical genetic methods have a more widespread application in drug screening.

Transgenic technology is the preferred method for epigenetic research and to develop more accurate disease models. Forward and reverse genetics have been used in zebrafish models, exploiting the fully sequenced genome and high-throughput sequencing (18). These approaches have provided a better view of the pathogenesis of IBD, which will be discussed in detail in the following section. The zebrafish model as drug screener is also a hot research topic. The key point is that providing specific exogenous substances to the transgenic zebrafish would lead to specific tissue changes associated with different quantities or qualities of a drug. For example, the TNBS-induced zebrafish model has been used to screen mesalazine (5-ASA) and prednisolone combined with immunofluorescence imaging (19).

Thus, zebrafish models show promise in IBD research in terms of pathogenesis and treatment. The important goals of using zebrafish models of IBD are determining and exploiting the pathogenesis of IBD, and reversing the growth and development of IBD in patients *via* drug screening or by immunosuppression.

## Advantages of Zebrafish Models

Investigators have used IBD animal models for over 100 years, from drug-induced methods to genetic engineering models (20). Although several models are similar to humans, none of them perfectly mimic the pathogenesis of IBD. In nature, enteritis can develop spontaneously in some animals, and these are termed spontaneous IBD models. Pathological changes analogous to UC found in the cotton-top tamarin (CTT) and the high morbidity rate of colon adenocarcinoma in adult CTTs make them an ideal model for UC-related colon carcinoma (21). However, their scarcity has hindered their use.

### General Comparison between Murine and Zebrafish

Murine models have gained popularity among IBD investigators. For instance, C3H/HeJ Bir mice were derived from C3H/HeJ mice with occasional enteritis (22). Pathological changes analogous to human CD are observed in the SAMP1/Yit mouse model, especially in the distal ileum (23). Although murine models have revealed the role of epithelial barrier function, the adaptive immune response and microbiota in the pathogenesis and susceptibility of IBD, and certain limitations, such as high cost, imaging limitations, and the difficulty of genetic manipulation techniques, have impeded their use in IBD research (24). In murine models, the pathological changes cannot be determined until the mice are killed, because the pathological development of IBD cannot be tracked in a direct and dynamic way (22).

### Cultivation and Breeding

Zebrafish are easier to cultivate than mice. Zebrafish are highly fecund: a female lays hundreds of eggs each time with a generation time of approximately 3–4 months and the eggs can be fertilized *in vitro*. It means zebrafish model can be established more efficient than traditional murine model. It is more suitable to the researches, in which large quantities of sample data are required. Besides, the optical transparency of the embryos and their rapid development facilitate observation, manipulation, and drug screening (25).

### Zebrafish Biology

The zebrafish intestine is a long tube composed of intestinal bulb, mid-intestine, and caudal intestine, which folds twice in the abdominal cavity (7). Three types of differentiated cells have been identified in the intestinal epithelium: absorptive enterocytes, goblet cells, and endocrine cells. The lamina propria beneath the gut epithelium contains macrophages and neutrophils (26). No Paneth cells, crypts, or submucosal glands have been detected in zebrafish (27, 28).

The immune system is the basis of the interaction between the host and microbes. The zebrafish immune system is highly analogous to that of humans. Adaptive immunity is not functional until 4 weeks after fertilization. Thus, during these 4 weeks, attention can be focused on the effects of innate immunity without the interference of adaptive immunity (24). According to current evidence, zebrafish have orthologs of mammalian toll-like receptors (TLRs), which are the innate immune receptors that recognize specific microbial molecules (29), and have functionally conserved orthologs of human IBD susceptibility genes *NOD1* and *NOD2* (11). Genetic techniques are easier to perform in zebrafish compared with mice. The improvement in genetic manipulation techniques for forward genetics has permitted the identification of over 160 IBD susceptibility genes in the human genome, most of which are associated with autophagy, host responses to bacteria, and the immune response: many of these have orthologs in the zebrafish genome (7, 24).

### Methods for Obtaining Results

Owing to its aqueous living environment, it is easier performing experiments where fish are exposed to chemicals or microbes in real time. In addition, aseptic techniques provide a convenient control of the microbial system in zebrafish (30). The rapid development of a series of bioscience techniques, including mutation techniques (insertional mutagenesis and ENU chemical mutagenesis), transgenic technology, and fluorescence labeling technique have accelerated the use of zebrafish as a new model system (31). Mutant strains of zebrafish are obtained to build models mimicking human disease or models for drug screening. One large-scale zebrafish genetic mutation research center, Cardiovascular Research Center, Massachusetts General Hospital, which preserves thousands of zebrafish mutants with embryonic or organ defects, is located in Massachusetts. This mutant collection will enable studies of zebrafish tissue or organ structure, morphology, function, metabolism, development, ethology, and pathological mechanism (25).

## Novel Insights in IBD Pathogenesis Using Zebrafish Models

Recently, IBD studies based on zebrafish models have focused mainly on five aspects: genes, development, interactions between gut microbiota and host immunity, and endoplasmic reticulum (ER) stress.

### Genetic Susceptibility

To date, 163 IBD susceptibility regions that were marked by single-nucleotide polymorphisms (SNPs) have been detected by genome-wide association studies (GWASs) in humans (32). Proteins and other functional molecules expressed in these regions have suggested the mechanisms of intestinal epithelial damage and defense, including epigenetic changes, the presence of endothelium stress, and the interaction between microbes and host immunity (33). The conservation of several core gut genes, such as *NOD1* and *NOD2*, in zebrafish and mammals makes zebrafish an interesting model to study the pathogenesis of intestinal inflammation and injury (11, 34). Although several studies have been performed based on the GWASs, their results may not be reliable. Heritability might be missing because the GWASs were poorly designed to reveal epistasis, which would underestimate the effects of non-coding regulatory regions (35). Thus, it is believed that the variants in protein-coding genes and non-coding DNA regulatory regions rich in SNPs might play a role in the pathogenesis (36).

Marjoram et al. developed a novel inflammation-responsive transgenic zebrafish line TgBAC (TNF-α: GFP), analysis of which confirmed the notion that IBD results from the loss of epigenetic repression and TNF overproduction in the intestinal epithelial cells. They used a forward genetic screen to identify mutants characterized by deficiencies in intestinal epithelial integrity (37). One of the mutants, named aa51.3^pd1092^, had a significantly disrupted intestinal epithelium. A mutation in ubiquitin-like protein including PHD and RING finger domains 1 (UHRF1), a highly conserved gene in methylation, was identified as the affected locus. The dysfunctional UHRF1 leads to hypomethylation of TNF-α promoter, which releases the transcription inhibition of the promoter, thereby leading to increased TNF-α in the intestinal epithelia. Increased TNF-α expression leads to the appearance of hallmarks of microbiota-dependent chronic inflammation, such as shedding and apoptosis of epithelial cells, immune cell recruitment, and barrier dysfunction (37). Therefore, this study identified a possible susceptibility gene for IBD. The unbiased study conducted by Marjoram and his colleagues encouraged other investigators to use GWASs or high-throughput sequencing to identify potential disease-associated genes. These studies could reveal novel genetic risk factors in non-coding regions and define their distinct functions in IBD pathogenesis.

Another genetic screen identified a mutant, cdipt^hi559^, which lacks phosphatidylinositol (PI) synthesis (38). Disturbance of PI signaling has been identified in gastrointestinal disease and inflammation (39). The cdipt^hi559^ mutants showed abnormal villous architecture and some features of IBD, including apoptosis of goblet cells, reduced mucus secretion, bacterial overgrowth, and leukocyte infiltration. Moreover, acute phase genes were upregulated and ER stress markers, such as hspa5 and xbp1, were activated strongly (40). This study identified a previously unknown link between intracellular PI signaling and ER stress-mediated gastrointestinal inflammation.

In addition, GWASs have revealed that SNPs close to *MST1* [encoding the macrophage-stimulating protein (MSP)] and *MST1R* [encoding the MSP receptor Ron (recepteur d’origine nantais)] were susceptibility factors of CD (41, 42). The identification of susceptibility factors in the MSP–RON signaling pathway implicates host responses to tissue lesions in CD. Current murine models with MSP and RON deficiency are incapable of uncovering the function of MSP–RON signaling pathway in intestinal inflammation. Nevertheless, positional cloning has led to the creation of a novel zebrafish *MSP* mutant (msp^t34230^) with a premature stop mutation (43). These zebrafish demonstrate intestinal eosinophilia, upregulated expression of the inflammatory marker MMP9 and goblet cell alterations. Additionally, ethanol-induced epithelial damage appears to be more severe in the MSP mutant. Intrarectal ethanol administration (20%) resulted in reduced survival, upregulated infiltration and proliferation of immune cells, and prolonged inflammatory cytokine responses in some MSP-deficient zebrafish (44). The innate cell recruitment and barrier dysfunction in the MSP and Ron mutants require further investigation to understand how disturbance of this pathway leads to chronic inflammation.

**Figure 2:**
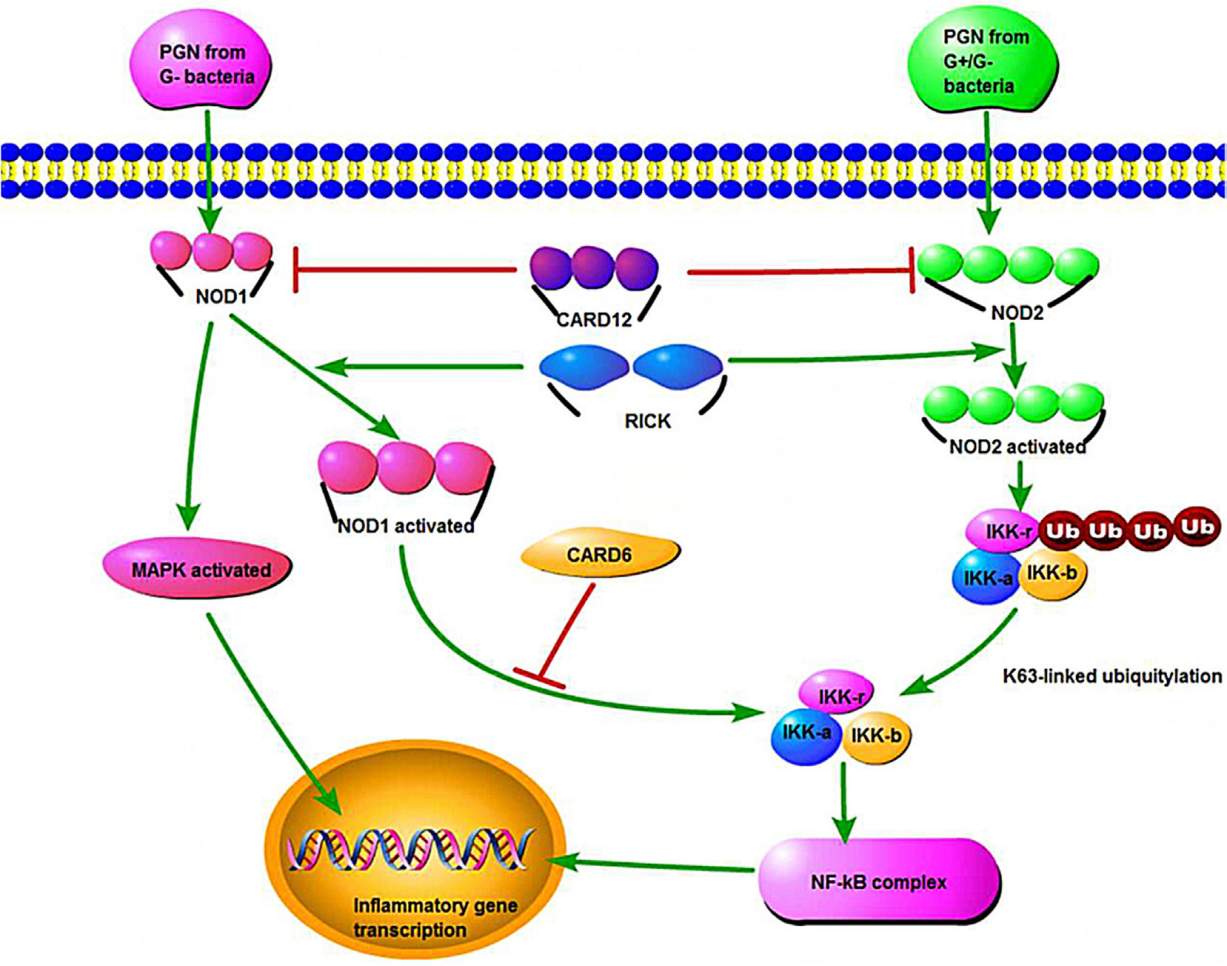
**The NOD1 and NOD2 signaling pathways, host defense, and inflammatory defense**. PGN represents peptidoglycan, and CARD12 is a member of the CED4/Apaf-1 family that can induce apoptosis. MAPK refers to mitogen-activated protein kinase, which stimulates the inflammatory process of cells. IKK refers to inhibitor of nuclear factor kappa-B (NF-κB) kinase, which participates in the cellular immune response caused by cytokines. NF-κB is a nuclear transcription factor that regulates the genes that are believed to be necessary in promoting inflammation.

While *NOD2* was the first human gene to be associated strongly with CD (45, 46), mutations in *NOD1* also show a disease-specific association with UC. *NOD1/2* is conserved in the human and zebrafish genomes and encodes intracellular bacterial sensor proteins that initiate the innate immunity (Figure 2) (11).

NOD signaling genes are expressed in intestinal epithelial cells and neutrophils. Morpholino oligomer (MO) knockdown is a useful tool to evaluate the zebrafish *NOD1/2* functions. In *NOD1/2* MO knockdown models, susceptibility to bacterial infections (like *Salmonella enterica*) increased and the expression of a dual oxidase that produces bactericidal reactive oxygen species was impaired (11).

### Immunology

The use of zebrafish to study IBD takes advantage of the combination of the growing list of immune cell reporter fish and the optical transparency of the larvae early in life (<2 weeks). Live imaging in zebrafish aids the study of intestinal immune cell function, the production of pro-inflammatory or anti-inflammatory cytokines, the interaction between the microbe and host immunity, and the integrity of the intestinal barrier (7). Hence, immune responses can be traced *in vivo* in a complete organism.

Several zebrafish models have demonstrated intestinal immune disorders in recent years. Chemically induced and genetic models have been established to determine the possible pathogenesis of IBD. Chemical zebrafish models have been induced by oxazolone, TNBS, and DSS (19, 20, 47–51). These models are characterized by the upregulated expression of cytokines, leukocytosis, and different degrees of morphological changes (19, 20, 48, 50).

Inflammatory bowel disease pathogenesis was studied initially in an oxazolone-induced enterocolitis zebrafish model. Brugman et al. administered the hapten oxazolone intrarectally to adult wild-type and myeloperoxidase-reporter transgenic zebrafish (20). They established an enterocolitis model successfully using histological and molecular evidence. Severe bowel-wall thickening, disappearance of the intestinal-fold architecture, depletion of goblet cells, and severe infiltration of eosinophils and neutrophils were observed. Oxazolone upregulated the expression of a series of genes encoding colitis-related cytokines, including pro-inflammatory (i.e., IL-1β, TNF-α) and anti-inflammatory cytokines (i.e., IL-10) in the intestine.

Another useful chemically induced zebrafish model was established by immersing the zebrafish in defined media mixed with TNBS. TNBS can induce intestinal inflammation and impairs gut functions in zebrafish larvae (50). Fleming reported morphological changes and increased numbers of goblet cells under exposure to 75 µg/mL TNBS (48). Oehlers documented that the same dose of TNBS exposure shortened the mid-intestine (segment II) and recruited neutrophils significantly in the intestine, but they did not observe significant intestinal morphological changes (19). Despite these differences, inflammation was confirmed by induction of the pro-inflammatory cytokines IL-1β and TNF-α, the degradative enzyme MMP9, and leukocytosis (19, 48). In addition to inflammation, loss of peristalsis and alteration of lipid metabolism have been documented (19, 48).

Dextran sodium sulfate has been used widely on mice and rats in studies of IBD pathogenesis (52). In zebrafish, DSS immersion is also useful to induce an enterocolitis model. Oheler reported the intestinal infiltration of neutrophils, global cell proliferation reduction, and upregulation of pro-inflammatory genes (i.e., TNF-α, IL-1β, IL-8, CCL20, and MMP9) after DSS exposure. Unlike the TNBS models, acidic mucins accumulated strongly in the intestinal bulb; however, the goblet cell number was relatively unchanged (50). The redundancy of mucins has a protective role in the gut epithelium during intestinal inflammation. The distinct mucin phenotype of the DSS zebrafish model permitted studies to uncover the regulation of mucins by various agents. For example, retinoic acid can suppress both basal and intestinal mucus production; thus exacerbating experimental enterocolitis in the zebrafish model (50).

Furthermore, using MO knockdown techniques, researchers can study the pathogenesis of IBD pathogenesis at the level of cytokines. Pro-inflammatory and anti-inflammatory cytokines maintain a dynamic balance of the immune response in the health body, and this balance is dysregulated in IBD patients.

Genome-wide association studies have proved that IL-23R is a susceptibility allele in IBD patients (26). IL-23 participates in the development of Th17 cells and the innate immune response (53). Using a synteny (conservation of gene order) approach, the IL-23 p19 subunit was identified in zebrafish. However, the exact function of IL-23 has not yet been determined. IL-23 is expressed constitutively in the zebrafish intestine and is induced by LPSs administration or *Mycobacterium marinum* infection (54). Hence, such a model could be used to study adaptive immunity in IBD pathogenesis.

IL-10 is an anti-inflammatory cytokine in the intestine. Lack of IL-10 might lead to IBD in humans (55). As a classic study object, IL-10 has been discussed a lot in the researches with murine models before. In recent years, with the warming up of zebrafish models, the researches of IL-10 can also been taken in the usage of zebrafish. IL-10 is conserved in the zebrafish intestine, and its expression was upregulated after LPS stimulation (56, 57). Using the zebrafish IL-10 mutant model, further study of IL-10 functions might increase our understanding of the host–microbe interactions and intestinal inflammation in IBD (58).

IL-22 is an immuomodulatory and multifunctional molecule in the intestine that is upregulated in CD patients (59). Studies in mice showed that IL-22 stimulates epithelial cells to produce antibacterial proteins, enhances mucus secretion, and strengthens epithelial regeneration (60). Studies using loss-of-function and gain-of-function approaches indicated that IL-22 could promote mucosal healing in acute intestinal injury. Recently, IL-22’s functions in intestinal inflammation in innate immunity were revealed (61). Silencing of *IL-22* increased the expressions of pro-inflammatory cytokines (IL-1β and TNF-α) in bacteria-stimulated fish. Further studies should focus on how this cytokine maintains the intestinal homeostasis in zebrafish.

In addition to the well-known pro-inflammatory and anti-inflammatory cytokines, inflammation-related factors and signaling pathway might also be associated with the pathogenesis of IBD. Zebrafish models could help investigators to study CXCL8, CCL13, and CCL14, which are difficult to study in murine models (62). For example, human CXCL8 regulates the chemotaxis of neutrophils. Unfortunately, murine models of CXCL8 deficiency are difficult to investigate. Surprisingly, the expression of CXCL8 is significantly enhanced in the larval intestine of TNBS-induced enterocolitis zebrafish models (49).

The heat shock proteins (HSPs) also have anti-inflammatory effects and can regulate intestinal immune function (63). Studies in mice showed that HSF1 (an HSP) and HSP70 play a protective role in IBD (64). Interestingly, the expressions of the HSP70 and HSP110 family genes *HSPa4* and *HSP4b* were increased in TNBS-induced enterocolitis zebrafish models (47).

Host responses to inflammation are dependent on the highly conserved neuropeptides of the brain–gut axis (64). Orthologs of corticotrophin-releasing hormone and urocortins (65), Substance P (66), neuropeptide Y (67), ghrelin (68), α-MSH (69), and gastrointestinal peptide (68) have been documented in zebrafish. The involvement of these neuropeptides in the host reaction to intestinal inflammation has been demonstrated in both murine and human studies (24, 70–75). Furthermore, an adult zebrafish model created by treatment with intrarectal injection of TNBS demonstrated that Melanin-concentrating hormone (MCH) expression is highly conserved among fish, mice, and humans (70). MCH is a conserved appetite-regulating neuropeptide that is implicated in the pathogenesis of IBD (71). The expression of MCH and the MCH receptor were upregulated in the intestine of model of zebrafish. Immunostaining revealed a reinforcement effect of colitis in zebrafish that was analogous to human IBD and TNBS-induced mouse colitis. However, the signaling pathways involved in zebrafish have not been determined (70).

Myeloid differentiation factor 88 (MYD88) acts as an adaptor protein in interleukin 1 receptor (IL-1R) and TLR signaling in human and mammalian models. Thus, MYD88 is one of the major molecules of the innate immune response. In a zebrafish model for signaling pathway studies, investigators first focused on the MYD88 signaling pathway. MYD88 is highly conserved in zebrafish (72, 73). This property of initiating the innate immune response makes MYD88 a response factor of the host–microbe interaction. Studies have revealed that the expressions of transcription factors (including NF-κB and AP-1) in the core of innate immunity and pro-inflammatory cytokines (including IL-1β, MMP9) depend on MYD88 signaling during bacterial infection. Acute bacterial infection induced by *Edwardsiella tarda* and *Salmonella typhimurium*, as well as chronic bacterial infections induced by *M. marinum*, induces severe intestinal lesions in zebrafish. MYD88-MO and MYD88 mutants had deficiencies of innate immunity and were increasingly susceptible to microbial infection (73). Moreover, knockdown of *MYD88* caused zebrafish to develop more severe intestinal lesions under TNBS exposure, suggesting that MYD88 has a protective role in pathogenesis of colitis. Hence, the MYD88 mutant zebrafish will be useful in studies of IL-1R and TLR signaling in the host–microbe interaction and in intestinal inflammation.

### Microbiota

Studies performed in gnotobiotic animals, such as mice and zebrafish, have demonstrated that the microbiota have an enormous impact on the host (74). The maturation of gnotobiotic techniques, particularly *in vitro* fertilization, has helped researchers to manipulate the species and to quantify the host–microbe interaction in zebrafish (24). Therefore, it has been suggested that zebrafish could be a more powerful animal model to study the aspects of the interaction between host and microbes.

To understand the effect of microbial colonization on the zebrafish host, Rawls et al. investigated gene expression changes in response to microbial colonization (75). The gene profiles demonstrated that microbial colonization altered the expressions of 212 genes. Among them, the responses of 59 genes were conserved between mice and zebrafish. These conserved genes were mainly involved in epithelial proliferation, promotion of nutrient metabolism, and innate immune responses. These data suggested that the response to microbes is highly conserved.

Experiments in zebrafish demonstrated that microbes influence fat absorption in the intestine. The presence of a microbiota resulted in increased fat storage in adipose tissue (76). Meanwhile, other studies showed that microbes stimulate fatty acid uptake in the intestinal epithelium and liver (77). These studies implied that diet-induced alterations of microbiota could lead to IBD by disturbing the host energy balance. Microbes can also induce intestinal development. Cheesman et al. documented that the presence of microbiota and Wnt signaling could stimulate epithelial cell proliferation during zebrafish intestinal development (78). Importantly, their findings demonstrated that homeostatic innate immune responses, but not inflammatory signals, contribute to the effects on epithelial proliferation. This could be explained by the fact that epithelial turnover depends on microbes *via* MYD88, but not *via* the TNF receptor (78). The absence of microbes hinders the differentiation of the intestinal epithelium (79). However, Bates et al. found several deficiencies in microbe-free intestines. There was a lack of brush border intestinal alkaline phosphate (IAP) activity and glycan expression was immature. The numbers of goblet cells and enteroendocrine cells decreased. Additionally, germ-free (GF) intestines failed to take up protein macromolecules in the distal intestine and presented a faster rate of peristalsis (79). Interestingly, colonization by microbes could completely reverse these GF manifestations. However, exposure to heat-killed microbes and LPS could restore the IAP activity, but not the glycan expression, suggesting that LPS induces IAP. IAP-deficient zebrafish are more sensitive to LPS exposure. Abundant neutrophils are observed under LPS exposure, while neutrophils are insufficient in the GF IAP-deficient phenotype. The phenotype of the IAP-deficient zebrafish was shown to depend on the proteins involved in LPS sensitivity in mammals (i.e., MYD88 and TNFR) (79). The above findings suggested that the presence of zebrafish intestinal endogenous microbiota maintains a normal level of neutrophils in the intestine through the involvement of three molecules: IAP, MYD88, and TNFR. Thus, IAP is involved in the protecting the intestine from microbe-related lesions and is suggested that abnormal microbiota cause IBD by influencing IAP activity.

Microbes intensify the host’s immune responses. The gut microbiota influences the host immune system through mutual interactions (74). In TNBS-induced enterocolitis zebrafish models, administration of ampicillin and kanamycin before TNBS in the fish media increased the survival of the fish and downregulated the expression of pro-inflammatory cytokines such as IL-1β, TNF-α, CCL20, and IL-8 (49). Studies in experimental animals have indicated that the intestinal microbiota play a critical role in the pathogenesis of intestinal inflammation (80). Dysbiosis potentially contributes to the pathogenesis of IBD by augmenting host pro-inflammatory immune responses.

Resident microbiota maintain the homeostasis of the host’s health (81). The composition of microbes in the host depends on the environment. Zebrafish are enriched in *Proteobacteria*, while mice and human have more *Bacteroidetes* and *Firmicutes* (75, 82). A study of intestinal microbiota dysbiosis in zebrafish induced by TNBS demonstrated an increased proportion of *Proteobacteria* and a decrease in the *Firmicutes* (83). Further analyses of this dysbiosis indicated a significant relationship with enterocolitis. Further studies confirmed the view that TLR signaling pathways recognize bacteria, resulting in the induced expression of immune molecules, including NF-κB and inflammatory cytokines (30). Investigators compared conventionally reared (CV) zebrafish, GF zebrafish, and TNBS-induced enterocolitis zebrafish models. TNBS exposure of CV zebrafish induced MYD88 and TRIF expression, NF-κB activation, and TNF-α expression. However, TNBS exposure of GF zebrafish and the control showed the absence of a TLR3 signaling pathway response and no detectable expression in MYD88 or TRIF, no NF-κB activation, or no TNF-α expression. Most importantly, TLR signaling pathways, including MYD88-related and MYD88-non-related pathways, appear to be the principal pathogenic pathways of enterocolitis.

In the oxalone-induced zebrafish model, the microbiota can influence the degree of enterocolitis. Brugman et al. established an enterocolitis score to evaluate inflammation. The score takes several factors into consideration, including the depletion of goblet cells, the infiltration of inflammatory cells and upregulation of pro-inflammatory cytokines (20). In vancomycin-treated zebrafish, the population of microbes was dominated by the *Fusobacteria*. In this model, decreased neutrophil infiltrations correlated with a reduced enterocolitis score. In the colistin sulfate-treated zebrafish model, the population of microbes was dominated by the *Proteobacteria*. Surprisingly, reduced eosinophil and lymphocyte infiltration was observed, while the enterocolitis scores remained unchanged. These findings indicated that different compositions of microbes determine the severity of enterocolitis and the composition of intestinal infiltration. Collectively, the results suggest that a deeper insight into chemokines and immune homologs in zebrafish is required.

### Epithelial Barrier Function-Related Reticulum Stress

Defects in the ER stress response and autophagy result in intestinal inflammation (84). Components of the ER stress response in the intestinal epithelium have been documented in IBD patients and murine models (85, 86).

The cdipt^hi559^ zebrafish mutant has a deficiency in PI synthesis and shows characteristics of ER stress in the intestinal epithelium (39). The abnormal intestinal morphologies comprise reduction of epithelial proliferation, apoptosis of epithelial and goblet cells, severe inflammation, and disruption of the normal bacteria–intestine balance. Two ER stress factors, HSPα5 and XBP1, are increased in the intestinal epithelium. Temporal electron microscopy analyses demonstrated that PI-deficient intestinal epithelium cells undergo chronic ER stress-mediated cytopathology, including disruption of the ER and Golgi apparatus, mitochondrial damage, autophagy, and apoptosis. Furthermore, the pharmacological induction of ER stress using tunicamycin to inhibit protein glycosylation or PI synthase inhibition in leukocyte-specific reporter lines produced similar phenotypes to those of the cdipt^hi559^ mutants. Antibiotics were administered to confirm the bacteria’s exacerbation of inflammation. In addition, chemical chaperones could reduce inflammation, indicating the therapeutic potential for small molecular agents in IBD (39).

In murine models, phospholipids showed a potential to resist inflammatory lesions (87). PI alleviates pathological ER stress, allowing the ER to perform its anti-inflammatory effect. Goldsmith et al. showed that the μ-opioid receptor agonist DALDA, a dermorphin analog (H-Tyr-d-Arg-Phe-Lys-NH_2_), alleviated ER stress to protect against intestinal injury in the glafenine-induced zebrafish model by inducing the unfolded protein response (UPR) (87). Investigators first established the zebrafish model by induction using non-steroidal anti-inflammatory drugs to simulate an impaired mucosal barrier function (88). The glafenine-induced phenotype was characterized by increased intestinal epithelial apoptosis and ER stress, but not by a reduction in the epithelial barrier function. Treatment with DALDA had no effect on the early UPR marker BIP, confirming the glafenine-induced effect. Surprisingly, the expressions of downstream proteins ATF6 and s-XBP1 did not increase, explaining the impaired UPR. These two studies clearly illustrated the feasibility of using zebrafish to study the barrier function in IBD.

### Developmental Biology

In developmental biology, fluorescence-activated cell sorting is combined with microarray profiling to identify genes and proteins involved in the intestinal development (89). The phosphatidylinositol 3 kinase pathway is implicated in this process and inhibition of it by LY294002 led to gastrointestinal defects. microRNAs (miR-217 and miR-122), tight junction protein claudin C, FAM136a, and zebrafish tetraspanin, are involved the process of gastrointestinal development. A protein complex containing claudin-7, the tetraspanin CO-029, and others in membrane microdomains has been identified (90). Investigators demonstrated that the claudin family member CLDNC and the tetraspanin LOC565274 are both preferentially expressed in the zebrafish gastrointestinal tract. The claudins participate in epithelial tight junctions and tetraspanins act on proteins in transmembrane signaling (91, 92). Dysregulation of these two proteins might lead to tumor metastasis and disruption of epithelial tight junction, contributing to the pathogenesis of IBD. Genes containing a putative transcription factor binding sequence, GGAANCGGAANY and a nucleolar gene network were identified in novel pathways, such as the PI3 signaling and inositol metabolism pathway, by activating the key mediator AKT2 (89). Multiple factors might tip the balance between intestinal homeostasis and inflammation (93). Thus, the complexity of cytokines and proteins maintains the normal functions of intestinal epithelial cells.

### Drug Screening

Different therapeutic methods can be developed according to the experimental evidence. Current pharmaceutical research is mainly focused on the microbiota or the host inflammation response. However, drugs to treat these facets of IBD will not achieve mucosal healing. Thus, continuous drug screening is needed to prevent adverse events, to substantiate treatment strategies, and to find innovative treatments (91).

The predominant advantage for TNBS-induced zebrafish models is consistency with high-throughput technology and the capacity to build a poly-phenotype library (93). Although there is no formal report of any subclinical human IBD medication tested to date, 5-ASA and prednisolone have been administrated and investigated for their pharmacodynamic responses. The effectiveness of these two widely used chemical treatments has been proved by evaluating the level of cytokines and the redistribution of leukocytes in intestinal regions of the inflamed larvae. Anti-inflammatory effects were observed for 2 days after administration, which was consistent with their curative effects in humans. This not only demonstrated the fidelity of the chemically induced zebrafish enterocolitis models to IBD but also the feasibility of using zebrafish to identify novel drugs for treating IBD (19).

## Deficiencies of Zebrafish

Despite their advantages, zebrafish have certain limitations. The immune system of the zebrafish has some unique characteristics compared with mammals. Duplicated genes are one of the essential parts of the zebrafish genome, which indicates that homologs of mammal immune factors might not have conserved functions in zebrafish. In addition, TLR4 of the zebrafish does not bind with LPS, which is the TLR4 ligand of mammals (44). In terms of anatomy, zebrafish have no lymph glands and their gut lacks Peyer’s patch. There are also differences between the zebrafish and mammals in the sites of T, B lymphocytes maturation and in subtypes of antibodies. For instance, B lymphocytes of zebrafish are produced in the kidney, while in mammals, they are produced in the bone marrow. Besides, researches of the stem cells located at the base of the crypt of mammalian intestinal epithelium are heating up in recent years (94). And these intestinal stem cells have also been proved to be significant drivers of epithelial homeostasis and regeneration (95). While the zebrafish intestinal epithelium is lack of crypts, which contributes to obvious limitation in the research of these popular field. In addition, the adaptive immune system does not develop until 4 weeks after fertilization; however, this delay benefits the study of innate immunity. For the intestinal microorganisms, those living in mammals do not survive in the zebrafish gut, making it difficult to study the regulation and function of adaptive immunity in host–microbe interactions (37). Moreover, current technology cannot produce sufficient nutritional information to permit the raising of GF zebrafish to adulthood.

## Conclusion and Perspectives

Despite some limitations, zebrafish are superior to the existing models of intestinal inflammation. The genetic and immunological similarities between zebrafish and humans mean that zebrafish are replacing mice as model organisms. With the assistance of newly developed strains, and high-throughput genetic and chemical screens, investigators are able to clarify the suppressors and the enhancers of inflammation (32). By contrast, the transgenic lines offer more unique features compared with other experimental platforms. In addition, we can take advantages of the similarities between the immune systems of zebrafish and humans (45). Zebrafish models can be used to determine whether the products of IBD susceptibility genes are pathogenic. Although chemically induced models construct an inflammatory environment, they do not match well with the criteria of IBD. Zebrafish is a low-cost experimental animal with a huge potential; however, the establishment and development of aseptic zebrafish farming require further investment.

## Author Contributions

Conception and design: SJ and LH. Acquisition of information; analysis and interpretation of information: LH, LX, CX, QY, FJ, and RZ. Writing, review, and/or revision of manuscript: SJ, LH, LX, CX, and QY. Format proofread: QY, LH, and SJ.

## Conflict of Interest Statement

The authors declare that the research was conducted in the absence of any commercial or financial relationships that could be construed as a potential conflict of interest. The reviewer, KL, and handling Editor declared their shared affiliation, and the handling Editor states that the process nevertheless met the standards of a fair and objective review.
